# Parallel Evolution of Genome Streamlining and Cellular Bioenergetics across the Marine Radiation of a Bacterial Phylum

**DOI:** 10.1128/mBio.01089-18

**Published:** 2018-09-18

**Authors:** Eric W. Getz, Saima Sultana Tithi, Liqing Zhang, Frank O. Aylward

**Affiliations:** a Department of Biological Sciences, Virginia Tech, Blacksburg, Virginia, USA; b Department of Computer Science, Virginia Tech, Blacksburg, Virginia, USA; University of Texas at Austin

**Keywords:** bioenergetics, candidate phyla, evolutionary genomics, genome streamlining, microbial oceanography, pangenomics

## Abstract

Diverse bacterial and archaeal lineages drive biogeochemical cycles in the global ocean, but the evolutionary processes that have shaped their genomic properties and physiological capabilities remain obscure. Here we track the genome evolution of the globally abundant marine bacterial phylum *Marinimicrobia* across its diversification into modern marine environments and demonstrate that extant lineages are partitioned between epipelagic and mesopelagic habitats. Moreover, we show that these habitat preferences are associated with fundamental differences in genomic organization, cellular bioenergetics, and metabolic modalities. Multiple lineages present in epipelagic niches independently acquired genes necessary for phototrophy and environmental stress mitigation, and their genomes convergently evolved key features associated with genome streamlining. In contrast, lineages residing in mesopelagic waters independently acquired nitrate respiratory machinery and a variety of cytochromes, consistent with the use of alternative terminal electron acceptors in oxygen minimum zones (OMZs). Further, while epipelagic clades have retained an ancestral Na^+^-pumping respiratory complex, mesopelagic lineages have largely replaced this complex with canonical H^+^-pumping respiratory complex I, potentially due to the increased efficiency of the latter together with the presence of the more energy-limiting environments deep in the ocean’s interior. These parallel evolutionary trends indicate that key features of genomic streamlining and cellular bioenergetics have occurred repeatedly and congruently in disparate clades and underscore the importance of environmental conditions and nutrient dynamics in driving the evolution of diverse bacterioplankton lineages in similar ways throughout the global ocean.

## Abstract (precis)

Understanding long-term patterns of microbial evolution is critical to advancing our knowledge of past and present role microbial life in driving global biogeochemical cycles. Historically, it has been challenging to study the evolution of environmental microbes due to difficulties in obtaining genome sequences from lineages that could not be cultivated, but recent advances in metagenomics and single-cell genomics have begun to obviate many of these hurdles. Here we present an evolutionary genomic analysis of the *Marinimicrobia*, a diverse bacterial group that is abundant in the global ocean. We demonstrate that distantly related *Marinimicrobia* species that reside in similar habitats have converged to assume similar genome architectures and cellular bioenergetics, suggesting that common factors shape the evolution of a broad array of marine lineages. These findings broaden our understanding of the evolutionary forces that have given rise to microbial life in the contemporary ocean.

## INTRODUCTION

Microbial life plays a central role in driving biogeochemical cycles in the ocean that have a critical impact on the broader chemical environment of Earth (1). Despite their importance, difficulties in laboratory cultivation have long hampered the analysis of ecologically important microbial groups, and cultivation-independent methods have become indispensable tools for studying microbes in the environment over the last ∼40 years (2, 3). Among the diverse cultivation-independent methods now in use, metagenomics and single-cell genomics have been applied widely, and several large-scale sequencing projects using these approaches have recently provided substantial advances in our understanding of microbial lineages that are abundant in the ocean (4–7).

Comparative genomic approaches have long been considered to be effective methods for studying environmental *Bacteria* and *Archaea* (8, 9). While the identification of functional marker genes and the reconstruction of metabolic pathways encoded in genomes often yield important insights into cellular physiology, analysis of genomic architecture and organization can provide clues to the ecological and evolutionary forces that have shaped microbial lineages through time. Early genomic studies using these approaches noted that the genomes of several globally abundant epipelagic bacterioplankton were small, compact, and relatively AT rich (10, 11), and later studies leveraging single-cell sequencing and metagenomic methods have confirmed the ubiquity of streamlined bacterial and archaeal genomes in the ocean (6, 12–15). These observations eventually led to the theory of genome streamlining, which posits that many abundant bacterioplankton lineages experience strong selective pressure for efficient nutrient usage in oligotrophic environments, which drives the evolution of compact genomes with short intergenic regions and few extraneous genes (16). More recent studies in nonmarine environments have continued to identify small, streamlined genomes, suggesting that these processes may be widespread across the biosphere (17, 18).

In this study, we present an evolutionary genomic analysis of the candidate phylum *Marinimicrobia*, which comprises a diverse group of microbial lineages that are abundant in the biosphere and for which no representative has yet been brought into pure culture and analyzed in the laboratory. The first studies of *Marinimicrobia* were performed using samples collected in the Sargasso Sea and in waters near the Oregon coast, where this group, also referred to as SAR406 or Marine Group A, was identified as a prevalent marine bacterioplankton lineage distantly related to the *Chlorobi* and *Fibrobacteres* (19). More-recent work has shown that members of this phylum can use a broad diversity of alternative electron acceptors in the ocean and likely play a central role in shaping biogeochemical cycles along environmental gradients (20). Moreover, other studies have shown that *Marinimicrobia* are present and active in a broad array of marine environments, including coastal and pelagic surface waters, cold seep brine pools, coastal “dead zones,” and oxygen minimum zones (OMZs), and likely mediate key transformations of nitrogen and sulfur throughout the global ocean (21–27). In contrast to the broad environmental distributions typical of other bacterial phyla, *Marinimicrobia* are unusual in that the vast majority of known diversity in this group has been observed in marine environments, thereby providing a unique opportunity for comparative genomic analyses to assess the factors shaping their genome evolution throughout their radiation into the contemporary ocean.

## RESULTS AND DISCUSSION

### Phylogenomics and biogeography of *Marinimicrobia*.

**FIG 1:**
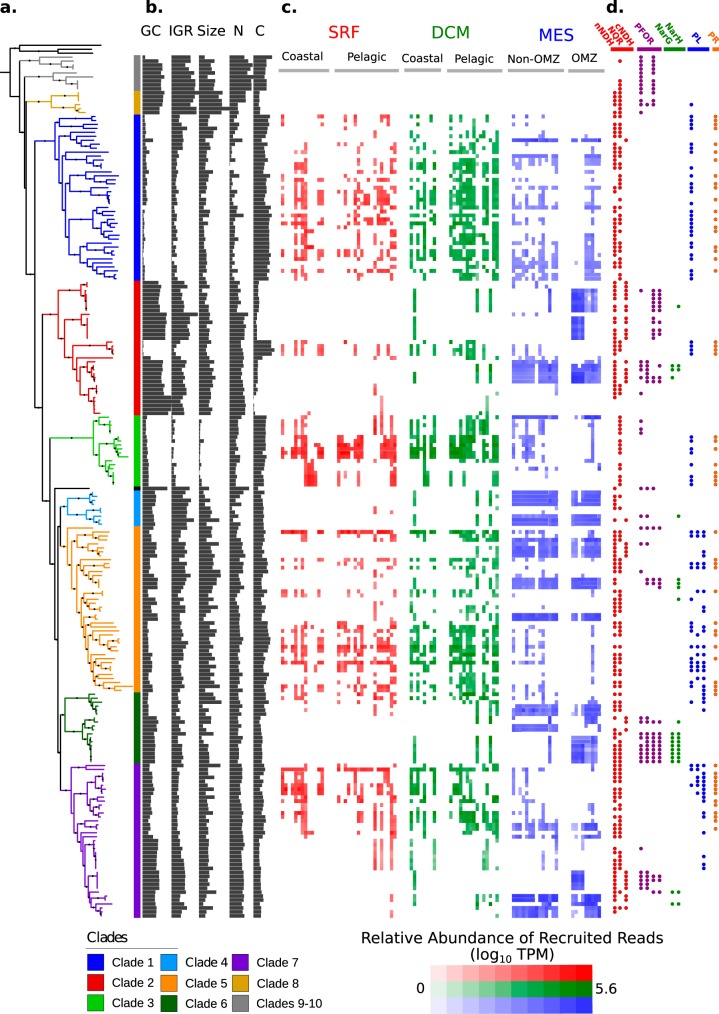
Overview of the phylogeny, genomic features, biogeography, and coding potential of the *Marinimicrobia*. (a) A phylogenetic tree of 218 *Marinimicrobia* genomes constructed using a concatenated alignment of 120 conserved marker genes. Prominent clades are colored, and nodes with support values of >0.95 are denoted with black circles. (b) Genomic features of the marinimicrobial genomes. Abbreviations: GC, % GC content (range, 30 to 50%); IGR, mean intergenic region length (range, 40 to 80 nucleotides [nt]); size, estimated genome size (range, 1 to 3.5 Mbp); N, N-ARSC (range, 0.3 to 0.34); C, C-ARSC (range, 3.2 to 3.4). (c) Heat map showing the abundances of marinimicrobial genomes in different ocean metagenomes. Abundances are in units of log_10_ TPM. Environmental features for the samples are the same as those provided by the Tara Oceans Consortium. Abbreviations: SRF, surface waters; DCM, deep chlorophyll maximum; MES, mesopelagic; OMZ, oxygen minimum zone. (d) Presence of selected bioenergetic complexes and marker genes in the marinimicrobial genomes. Abbreviations: PL, photo-lyase; PR, proteorhodopsin. See main text for details.

We compiled a set of 218 publicly available partial marinimicrobial genomes that had been generated using single-cell or metagenomic approaches (5, 20–22, 28–30) (see Materials and Methods). Our phylogenetic analysis of these genomes using concatenated amino acid alignments of marker gene sequences yielded 10 major clades that encompass the majority of known diversity in this phylum (Fig. 1a; see also Fig. S1 and S2 at figshare.com/projects/Marinimicrobia_Pangenomics/30881). Through comparison of this phylogeny with our genome abundance estimates from Tara Oceans samples (4), we identified seven clades (clades 1 to 7) that belong to a single monophyletic group and that are prominent in planktonic marine ecosystems around the globe (Fig. 1c). The remaining three basal branching clades (clades 8 to 10) appear to have more-restricted biogeographic distributions that include methanogenic bioreactors (29), deep sea brine pools (22), and oil reservoirs and fields (31). The structure of the tree is therefore consistent with a radiation of the *Marinimicrobia* that took place at the base of clades 1 to 7, with subsequent lineages diversifying into coastal and pelagic planktonic niches throughout the global ocean. Given that clades 1 to 7 contained the majority of marinimicrobial genomes, appeared more prevalent in global ocean waters, and exhibited a well-defined biogeography, we focused our subsequent analyses on these clades.

**FIG 2:**
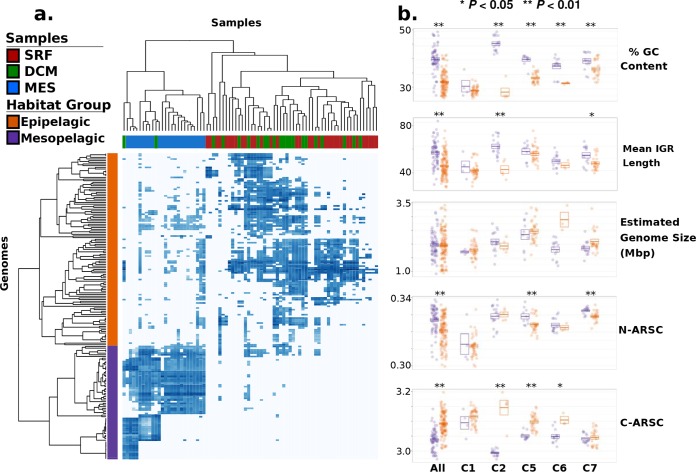
Habitat-based groupings of marinimicrobial genomes and their genomic features. (a) Heat map showing the abundance of marinimicrobial genomes in clades 1 to 7 in different metagenomic samples, with both samples and habitat groups color coded. Note that the abundance values here are the same as those presented in Fig. 1c (units of log_10_ TPM). (b) Dot plots showing the genomic features of *Marinimicrobia* genomes between habitat groups (orange, epipelagic; purple, mesopelagic). Each dot represents a genome, and boxes indicate the means and standard errors. Asterisks denote significant differences between epipelagic and mesopelagic genomes (**, *P*  < 0.01; *, *P*  < 0.05). The “All” category includes all genomes in clades 1 to 7 that could be assigned to a habitat group, while categories C1, C2, C5, C6, and C7 show only the genomes corresponding to those clades. Clades 3 and 4 are not shown since they did not include multiple genomes in both habitat groups.

We observed that *Marinimicrobia* in clades 1 to 7 were predominantly present in either epipelagic or mesopelagic waters but not in both, consistent with previous findings of distinct structuring of oceanic microbial communities by depth (32–35) (Fig. 1a and c). We confirmed this finding by clustering genomes of clades 1 to 7 according to their biogeographic distributions and recovering two major habitat groups that correspond to genomes found in epipelagic or mesopelagic waters (Fig. 2a). This pattern of habitat preference is exemplified clearly in clade 2 (red in Fig. 1), in which the basal branching lineages are present in mesopelagic waters, and one derived subclade appears to have switched to the epipelagic habitat. Genomes in clade 3 were found almost entirely in surface waters; genomes in clade 4 were found almost entirely in mesopelagic waters; and genomes in clades 1, 2, 5, 6, and 7 contained several genomes that were found in both environments (Fig. 1a and c).

### Parallel evolution of genome streamlining in *Marinimicrobia*.

We found that the habitat preferences observed throughout the marinimicrobial tree were strongly correlated with patterns of genomic organization. Genomes of epipelagic *Marinimicrobia* exhibited signatures of streamlining such as lower percent GC content and shorter intergenic regions (16, 36) (Fig. 1a and c and 2b). Moreover, epipelagic *Marinimicrobia* also exhibited fewer nitrogen atoms per residue side chain (N-ARSC) in their encoded proteins, consistent with the hypothesis that this represents an adaptation to reduce nitrogen demand in oligotrophic surface waters (32, 37). In contrast, mesopelagic *Marinimicrobia* contained lower carbon content in their encoded proteins (C-ARSC), consistent with higher nitrogen but lower carbon availabilities in deeper waters (32) (Fig. 2b). Many of these features were correlated, suggesting the presence of distinct genomic modalities in epipelagic versus mesopelagic *Marinimicrobia* (see Fig. S3 at figshare.com/projects/Marinimicrobia_Pangenomics/30881), which is consistent with observations of a genomic transition zone between these two regions (32).

Overall, we found GC content, mean intergenic spacer length, N-ARSC, and C-ARSC to be significantly different between all *Marinimicrobia* in the two habitat categories (Mann-Whitney U test, *P  < *0.01, “All” category in Fig. 2a), and our intraclade comparisons demonstrate that these disparities evolved independently in several different clades (Fig. 2b). Percent GC content was the most prominent feature that shifted with habitat preference, with clades 2, 5, 6, and 7 all showing significantly lower values in epipelagic versus mesopelagic genomes. C-ARSC was the next most prevalent feature distinguishing between habitat groups, with 3 clades showing significant differences. Interestingly, clade 1 did not show genome features that were significantly different between groups despite the epipelagic genomes in this group displaying marked indications of streamlining (Fig. 1a and b). This is likely because only 5 genomes in this clade are more abundant in mesopelagic waters, which limits the statistical power of comparisons. Moreover, the genome with the highest GC content, second highest N-ARSC, and lowest C-ARSC in clade 1 belongs to *Marinimicrobia* NORP180, which is the genome in this clade found to be most abundant in the mesopelagic habitat (Fig. 1), suggesting that genomic transitions have begun to evolve in this lineage. Other lineages that may have recently switched between habitats may have not had enough time to acquire the genomic features typical of their new environment, indicating that these traits require long periods of time to evolve.

Although C-ARSC and N-ARSC are not typically considered indicators of genome streamlining, our findings indicating that these metrics vary consistently with other aspects of streamlined genomes in *Marinimicrobia* suggest that they represent salient features that future studies should consider when assessing bacterioplankton genome evolution. Recent analysis of whole-community genomic differences between epipelagic and mesopelagic microbes has also shown that C-ARSC and N-ARSC are strongly connected to other features associated with streamlining, such as percent GC content and mean intergenic region length (32). The evolution of efficient nutrient utilization strategies is an important aspect of streamlining theory (16), and because proteins comprise a large pool of cellular carbon and nitrogen, it is likely that shifts in N-ARSC or C-ARSC that aid in the efficient allocation of macronutrients are highly advantageous in oligotrophic environments. Recent modeling of genome evolution in marine bacteria has indicated that changes in nutrient allocation can exert a strong influence on other genomic features such as percent GC content (38).

Interestingly, we did not observe a consistent reduction in genome size in epipelagic versus mesopelagic *Marinimicrobia* (Fig. 2b), which is perhaps paradoxical, considering that the genomes in the former group appear more streamlined in most other aspects. We would expect that the genomes of epipelagic *Marinimicrobia* would experience at least a modest decrease in genome size due to their shorter intergenic regions, but this reduction may be minor and not statistically significant given that coding regions comprise the vast majority of total DNA. The lack of large differences in genome size between epipelagic and mesopelagic *Marinimicrobia* is not entirely surprising given that streamlining has been hypothesized to take place over a range of genome sizes, since adaptation to a given environment requires particular coding potential that would in turn dictate genome size (16). Our results are consistent with this hypothesis and suggest that in the genome streamlining that we observed in the *Marinimicrobia*, which appears to be driven largely by differential selection in distinct environments and nutrient regimes, we would not necessarily expect to observe large differences in genome size but rather changes in features such as intergenic spacer length, percent GC content, and N-ARSC and C-ARSC, for which we saw strong and repeated shifts.

### Convergence of functional repertoires in *Marinimicrobia*.

**FIG 3:**
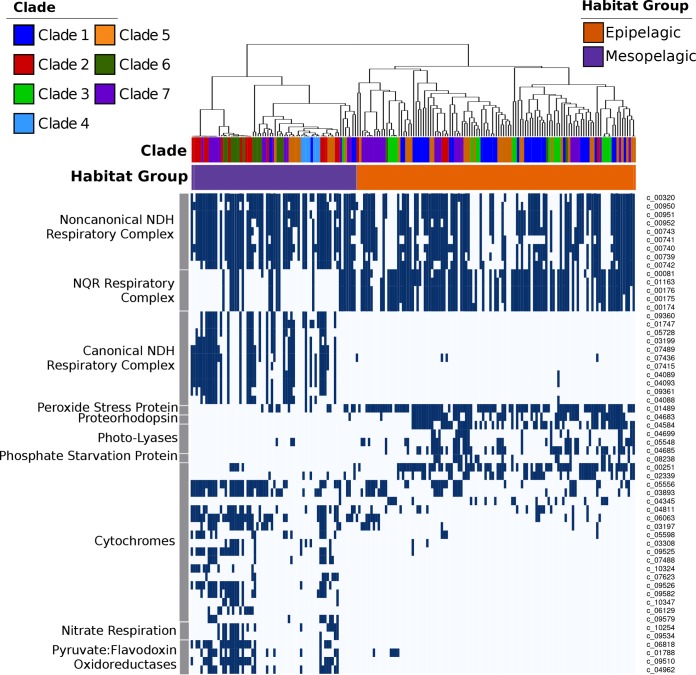
Presence of selected marker genes and bioenergetic complexes across the *Marinimicrobia*. The dendrogram on top shows the habitat-based genome clustering, and the color strips below it show the habitat groups and clades of the genomes. The colors used to denote habitat groups and clades are identical to those in Fig. 1 and 2. Unique identifiers for the protein clusters are indicated on the right.

**FIG 4:**
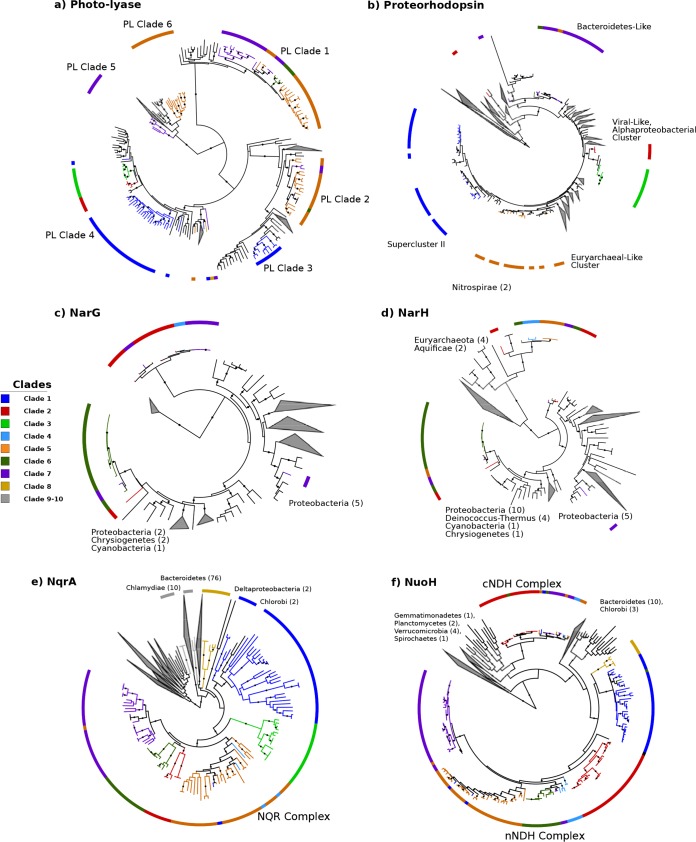
Evolutionary history of metabolic marker genes in the *Marinimicrobia*. Phylogeny of marinimicrobial photolyases (COG0415; panel a), proteorhodopsins (ENOG4111G9N; panel b), NarG (COG5013; panel c), NarH (COG1140; panel d), NuoH (COG1005; panel e), and NqrA (COG1726; panel f). Each phylogeny also contains reference sequences, which were obtained from the EggNOG website for all phylogenies except those of proteorhodopsins, which were obtained from the MicRhoDE database (40) (see Materials and Methods). Solid circles denote nodes with support values of >0.95. Interactive phylogenies are available online at http://itol.embl.de/shared/faylward.

We also identified clear differences in genomic repertoires between epipelagic and mesopelagic *Marinimicrobia*, with our pangenomic analyses revealing 758 orthologous groups that were enriched in either of the habitat groups (Fisher’s exact test; corrected *P* values of <0.01). Many epipelagic *Marinimicrobia* in clades 1 to 7 have acquired proteorhodopsin proton pumps, photolyases associated with UV stress, peroxide stress genes, and phosphate starvation genes, consistent with convergence toward similar mechanisms for life in oligotrophic surface waters in which UV radiation, peroxides, and low nutrient levels are prevalent stressors (39) (Fig. 3). Our phylogenetic analysis of marinimicrobial photolyases and proteorhodopsins indicated that these genes were acquired independently in different clades, supporting the hypothesis of parallel evolution of distantly related *Marinimicrobia* species toward similar ecological niches (Fig. 4a and b). In contrast, several mesopelagic *Marinimicrobia* genomes have independently acquired the cellular machinery for nitrate respiration (Fig. 1d and 4c and d), mirroring the independent gene acquisitions observed in epipelagic groups and consistent with findings that many *Marinimicrobia* are poised to exploit alternative electron acceptors under conditions of low oxygen concentrations (21, 41).

We also identified several cytochrome-associated proteins and pyruvate:ferredoxin/flavodoxin oxidoreductases (PFORs) that were differentially enriched in epipelagic versus mesopelagic *Marinimicrobia* (Fig. 3; see also Table S1 at figshare.com/projects/Marinimicrobia_Pangenomics/30881), with all PFOR subunits and most cytochrome subunits more prevalent in mesopelagic groups. Recent work has shown that cytochrome *c* oxidases are coexpressed with anaerobic respiratory genes in some *Marinimicrobia* under conditions of low levels of dissolved oxygen, suggesting that these cytochromes are either involved in the coreduction of electron acceptors other than oxygen or involved in the rapid switching between aerobic and anaerobic metabolism (21). Another study of microbial communities in a subseafloor aquifer that included *Marinimicrobia* also identified genomic signatures of anaerobic respiration despite oxic conditions (41), further suggesting that switching between electron acceptors may be a dynamic process in deep marine environments that is dictated by prevailing environmental conditions. Overall, the presence of a wide array of cytochromes in mesopelagic *Marinimicrobia* is consistent with their use of a variety of terminal electron acceptors, which is similar to what has been observed in other well-studied microbes such as Shewanella oneidensis (42). The prevalence of PFORs in mesopelagic *Marinimicrobia* is potentially consistent with the metabolic versatility of these bacteria, since the ability to shuttle electrons through alternative carriers such as ferredoxin or flavodoxin may allow a broader range of respiratory complexes to be used. Our phylogenetic analyses of PFORs are consistent with multiple independent acquisitions by mesopelagic *Marinimicrobia* (see Fig. S4 at figshare.com/projects/Marinimicrobia_Pangenomics/30881), further indicating convergence toward similar bioenergetic modalities among habitat groups.

Perhaps most strikingly, the different evolutionary forces experienced by epipelagic and mesopelagic *Marinimicrobia* also appear to have altered their cellular bioenergetics, as we observed a prevalence of NQR-(Na^+^) respiratory complexes in epipelagic *Marinimicrobia*, while mesopelagic groups appear to have largely replaced this with a canonical NDH-(H^+^) respiratory complex (cNDH) (Fig. 1d and 3). Most genomes in both groups also encoded a noncanonical NDH-(H^+^) respiratory complex (nNDH) for which the NADH reductase subunits were missing, suggesting that alternative electron donors such as flavodoxin may be used, which is similar to what has been observed in other groups (43). These findings indicate that while use of both a sodium motive force and a proton motive force is prevalent across *Marinimicrobia*, the relative levels of importance of these bioenergetic gradients and how they are used differ between groups. There are a number of possible explanations for these differences between epipelagic and mesopelagic *Marinimicrobia*. First, the canonical NDH-(H^+^) complex is likely more efficient than the NQR-(Na^+^) pump (44), which is potentially favorable to mesopelagic *Marinimicrobia* since carbon and energy are less readily available deeper in the water column. For epipelagic *Marinimicrobia*, an NQR respiratory complex may be sufficient given that nitrogen and phosphorus availabilities and environmental stressors more often limit growth for these bacterioplankton than energy availability. Second, epipelagic waters have slightly higher pH and salinity (45), which may create a more favorable environment for the harnessing of a sodium motive force in surface waters and a proton motive force deeper in the water column. Last, it is possible that the reactive oxygen species (ROS) produced by NDH make the use of this complex disadvantageous in surface waters, where high hydrogen peroxide concentrations already generate substantial quantities of these stressors (46), though it is unclear if the NQR complex of *Marinimicrobia* produces fewer ROS.

A combination of vertical inheritance and lateral gene transfer (LGT) appears to have shaped the distribution of respiratory complexes throughout the *Marinimicrobia*. The NQR complex is prevalent throughout the *Marinimicrobia* phylogeny, including basal branching clades 9 and 10, suggesting that this complex was present in the common ancestor of all *Marinimicrobia* (Fig. 1d). Phylogenetic analysis of NqrA revealed a topology similar to that of the main *Marinimicrobia* clades, further suggesting that the NQR-(Na^+^) complex was present in the ancestral *Marinimicrobia* and has evolved primarily through vertical inheritance (Fig. 4e). The nNDH complex also appears broadly represented in *Marinimicrobia*, but its absence in basal branching groups 9 and 10 suggests that this complex either was acquired at the last common ancestor of clades 1 to 8 or was present in the last common ancestor of all *Marinimicrobia* and was then subsequently lost in clades 9 and 10 (Fig. 4f). The evolutionary history of the nNDH complex is broadly consistent with the marinimicrobial phylogeny, consistent with both of these scenarios. The distribution of the cNDH respiratory complex is the most restricted, with only selected mesopelagic *Marinimicrobia* harboring the gene cluster. Moreover, phylogenetic analysis of the NuoH subunit in cNDH revealed a phylogeny inconsistent with the *Marinimicrobia* phylogeny, suggesting that LGT is largely responsible for shaping the distribution of this gene cluster across the phylum. Among the members of the clade of cNDH NuoH proteins, clade 2 appears to have the most divergent sequences, suggesting that this gene cluster may have been acquired from the common ancestor of clades 2 to 7 and then transferred between clades afterward (Fig. 4f).

### Conclusion.

Our combined assessment of the evolutionary genomics and biogeography of the globally abundant candidate phylum *Marinimicrobia* has revealed a pattern of parallel genomic, metabolic, and bioenergetic transitions that have occurred in multiple clades concomitant with their shifts between epipelagic or mesopelagic habitats. The large number of features that have converged in disparate clades is surprising, and it suggests that strong selective pressure leads to reproducible and in some ways predictable outcomes in diverse bacterioplankton. Moreover, it provides a link between disparate traits such as cellular bioenergetics and genome organization that are not typically considered to be strongly correlated in microbial life. The breadth of these findings across the *Marinimicrobia* suggest that similar trends in genome evolution are present in other bacterioplankton groups, though the extent likely varies depending on the specific habitat and the length of time that a given lineage has resided there. The *Marinimicrobia* are an ideal group to study these evolutionary trends because they represent a broad swath of phylogenetic diversity that is almost exclusively present in marine ecosystems, permitting the analysis of long-term trends in diversification within the same environment such as would not be present for other phyla of *Bacteria*.

Streamlining in epipelagic *Marinimicrobia* appears to be consistent with selection for increased nutrient allocation efficiency, which is a component of the initial formulation of streamlining theory (16). This selective pressure may result in numerous genomic changes; for example, because percent GC content and N-ARSC are correlated in the genetic code (47), it is likely that selection to decrease cellular nitrogen content leads to decreases in both percent GC and N-ARSC values. Moreover, selection for minimal nutrient allocation to DNA could result in shorter intergenic regions. In contrast, mesopelagic *Marinimicrobia* did not display features consistent with streamlining, but they did have significantly lower C-ARSC values in their encoded proteins, consistent with the importance of carbon limitation in driving selection in waters below the photic zone. It should be noted that we have considered mainly selective forces here, but recent work has suggested that genetic drift may also play a considerable role in genome streamlining (36, 48), and we cannot presently rule out the possibility that drift has also played a part in shaping the patterns of marinimicrobial genome evolution described here.

The lack of genome streamlining in basal branching *Marinimicrobia* suggests that streamlining is a derived feature that evolved independently in multiple distinct lineages after the divergence of clades 1 to 7. Moreover, epipelagic and mesopelagic *Marinimicrobia* from disparate clades independently acquired genes necessary for life in their respective habitats, including the notable acquisition of the cNDH complex in mesopelagic groups, indicating that parallel evolutionary trends have occurred in disparate lineages of both epipelagic and mesopelagic *Marinimicrobia*. Without detailed knowledge of ancestral genomes in this phylum, however, the exact sequence of evolutionary events remains unclear, and future work focusing on reconstructions of ancestral states may be helpful in clarifying the genomic repertoires and transitions of ancient *Marinimicrobia*.

In addition to providing insight into the ecological forces that shape this abundant and globally distributed bacterioplankton lineage, these genomic, metabolic, and bioenergetic transitions also provide a living record of the evolutionary processes that have given rise to extant *Marinimicrobia* throughout their diversification in the modern ocean. Continuing to establish the evolutionary processes that have shaped extant marine microbial groups is critical given that climate change and other more localized anthropogenic disturbances are changing global ocean ecosystems and biogeochemical cycles at an unprecedented rate. For example, both oxygen minimum zones and oligotrophic surface waters in oceanic gyres have been expanding due to climate change (49, 50). In the case of OMZs, many *Marinimicrobia* appear to have already evolved over millions of years to use alternative electron acceptors and may therefore be poised to exploit these expanding ecological niches. How shifts in global biogeochemistry will in turn change the ecological and evolutionary trajectories of microbial life is unknown, but establishing the evolutionary drivers that have given rise to the patterns of microbial diversity in the contemporary ocean is a critical first step toward being able to predict the outcome of future changes.

## MATERIALS AND METHODS

### Compilation of the *Marinimicrobia* genome set and phylogenetic reconstruction.

To compile a preliminary *Marinimicrobia* data set, we downloaded all genomes from GenBank that were annotated as belonging to the *Marinimicrobia* phylum according to the NCBI Taxonomy database (51) on 15 October 2017. Additionally, we supplemented the data sets with previously published genomes available in the Integrated Microbial Genomes system (IMG [52]) and from two recent studies that generated a large number of metagenome-assembled genomes (MAGs) (30, 53). For the study by Tully et al., we initially considered all genomes classified as *Marinimicrobia* as well as all genomes not given a classification. We used CheckM to assess the completeness and contamination of the genomes (54) and continued to analyze only those with contamination levels of <5% and completeness levels of >40%.

To confirm that all of the genomes were correctly classified as *Marinimicrobia*, we constructed a preliminary multilocus phylogenetic tree of all genomes using concatenated alignments of phylogenetic marker genes. To ensure that genomes from phyla closely related to *Marinimicrobia* were not being erroneously included in this analysis, we also included a variety of outgroup genomes from lineages known to be present in the same proximal location as *Marinimicrobia* in the tree of life (28), which included the phyla *Chlorobi*, *Bacteroidetes*, *Ignavibacteriae*, *Calditrichaeota*, *Fibrobacteres*, *Gemmatimonadetes*, *Latescibacteria*, *Zixibacteria*, and *Cloacimonetes* as well as the candidate phyla TA06, UBP1, UBP2, UBP11, WOR-3, and Hyd24-12. For initial phylogenetic assessments, we constructed a phylogenetic tree using the CheckM bacterial marker set (120 genes [54]), which we refer to here as the checkm_bact set. We predicted proteins using Prodigal v2.6.2 (55) and annotated the protein predictions from each genome through comparison to previously constructed hidden Markov models (HMMs) using HMMER3 with the recommended cutoffs previously reported (54). The scripts we used for this are publicly available on GitHub (github.com/faylward/pangenomics/). For alignment and phylogenetic reconstruction, we used the ETE Toolkit with the standard_trimmed_fastree workflow (56), which employs ClustalOmega for alignment (57), trimAl for alignment trimming (58), and FastTree for phylogenetic inference (59). The final tree can be viewed in Fig. S1 at figshare.com/projects/Marinimicrobia_Pangenomics/30881 and via a link to the interactive Tree of Life (iTOL [60]) (http://itol.embl.de/shared/faylward). Upon analysis of this tree, we removed three additional genomes (TOBG_SP-359, TOBG_MED-784, and TOBG_RS-789) from further analysis because they did not group with other *Marinimicrobia*. Additionally, to avoid unnecessary redundancy, we removed 10 MAGs because they had phylogenetic placements identical to those seen with other MAGs generated from the same metagenomic data. In those cases, the MAG with the highest estimated completeness was retained. Ultimately, we arrived at a final set of 218 *Marinimicrobia* genomes that we used in subsequent analysis. To construct a final tree, we used the checkm_bact marker gene set and the standard_trimmed_fasttree workflow of the ETE Toolkit, with the genomes of Fibrobacter succinogenes S85, Flavobacterium psychrophilum FPB101, and Bacteroides fragilis YCH46 as outgroups. This tree can be viewed in Fig. 1 and via interactive link on iTOL (http://itol.embl.de/shared/faylward). We identified major clades of *Marinimicrobia* through visual inspection of this final tree. Detailed information for all genomes used in this study can be found in Data Set S1 at figshare.com/projects/Marinimicrobia_Pangenomics/30881.

### Calculation of genomic characteristics.

We predicted GC content, N-ARSC, C-ARSC, and mean intergenic space length data using previously described methods (32). Code for these analyses is available online (https://github.com/faylward/pangenomics/). To estimate genome size (*S*), we used the following formula:

$$S=\frac{\alpha \left(1-\beta \right)}{\text{γ}}$$

where α is the number of base pairs in the genome assembly, β is the estimated level of contamination, and γ is the estimated level of completeness. We estimated contamination and completeness for each genome using CheckM v1.0.7 (54).

### Protein cluster identification and annotation.

We predicted proteins from all genomes using Prodigal and subsequently identified protein orthologous groups (OGs) using proteinortho v5.16b with default parameters (61). For each OG, we chose the longest member as a representative and compared these proteins to those in the EggNOG release 4.5 (62), Pfam release 31 (63), and TigrFam release 15.0 (64) databases for annotation using HMMER3 (65). For EggNOG, we downloaded all NOG HMMs from the EggNOG website on 1 February 2018 and ran hmmsearch with an E value cutoff of 1e−5. For Pfam and TigrFam annotations, we used the noise cutoffs in each HMM as the lower bounds for annotation.

### Respiratory complex annotation.

We annotated the NDH (H^+^) respiratory complex in a manner broadly similar to that previously reported (66). The canonical NDH (H^+^) respiratory complex consists of 14 subunits (*nuoA* to *nuoN* [*nuoA-N*]) that correspond to the COG HMMs COG0838, COG0377, COG0852, COG0649, COG1905, COG1894, COG1034, COG1005, COG1143, COG0839, COG0713, COG1009, COG1008, and COG1007. The subunits are usually syntenic, with the exception of *nuoN* and *nuoM*, which can sometimes be found on a distant chromosomal region or adjacent to other respiratory complexes. We identified protein OGs that corresponded to these COGs and considered a genome to contain this complex if at least 6 of the *nuoA-L* genes were present. We identified a second NDH respiratory complex in many genomes that lacked subunits D to F, and OGs corresponding to these subunits were distinct from those of the canonical NDH complex. We considered this second NDH complex to be present if at least 5 of the OGs corresponding to the *nuoABCGHIJK* subunits could be identified. For simplicity, we refer to the canonical *nuo* complex as cNDH and the noncanonical version lacking *nuoDEF* as nNDH. The canonical NQR-(Na^+^) respiratory complex consists of the 5 *nqrA-F* subunits, which correspond to COG HMMs COG1726, COG1805, COG2869, COG1347, COG2209, and COG2871. We identified OGs that corresponded to those COGs and considered a genome to encode the NQR complex if at least 3 OGs were present. Detailed information for each protein OG and their annotations and on which genomes encoded members can be found online (figshare.com/projects/Marinimicrobia_Pangenomics/30881).

### Marker gene phylogenies.

To assess the evolutionary histories of key marker genes in the *Marinimicrobia*, we constructed phylogenies with these genes together with available reference sequences. For each marker gene, we identified the NOG to which the OG had been annotated using our EggNOG annotations and then downloaded all proteins belonging to the appropriate NOG on the EggNOG website (62). The one exception to this was the procedure used for the proteorhodopsin phylogeny, for which we used the reference sequences available on the MicRhoDE database (40). Because the reference protein data sets were quite large, we reduced their size by clustering similar proteins using CD-HIT (67) (default parameters). These reference proteins were then combined with the *Marinimicrobia* proteins into a single FASTA file, and phylogenies were constructed using the ETE Toolkit (56), with the standard_trimmed_fasttree workflow. We refer to these as the “full phylogenies” since they included a large number of reference sequences. For ease of visualization, we manually selected a subset of reference sequences together with all *Marinimicrobia* sequences from the full phylogenies and then constructed smaller “subset phylogenies.” Subset phylogenies, with *Marinimicrobia* proteins colored by clade, are provided in Fig. 4 (see also Fig. S4 at figshare.com/projects/Marinimicrobia_Pangenomics/30881). Full phylogenies are available as interactive trees at http://itol.embl.de/shared/faylward.

### *Marinimicrobia* genome distributions and habitat distinctions.

To identify the biogeographic distributions of different *Marinimicrobia* genomes in global ocean samples, we downloaded 90 metagenome samples from the Tara Oceans expedition and mapped metagenomic reads against the final set of 218 *Marinimicrobia* genomes. We chose Tara Oceans samples to represent as broad a sampling of environments as possible and to include different depths (surface, deep chlorophyll maximum, and mesopelagic), ocean basins, and Longhurstian provinces. Details for the samples chosen are available online (figshare.com/projects/Marinimicrobia_Pangenomics/30881). We mapped reads using FastViromeExplorer (68), which, although initially intended for identification of viral sequences, includes a rapid and versatile read-mapping utility which contains built-in filters to remove spuriously identified sequences. We report final genome quantifications using the TPM (transcripts per kilobase million) metric (69), which corrects for sample size and reference genome length.

Habitat groups of *Marinimicrobia* were determined by hierarchical clustering of genome abundances in the Tara Ocean samples. For genome clustering, we loaded a log_10_-transformed genome TPM abundance matrix into R and calculated pairwise Pearson correlation coefficients for the genomes using the “cor” function. We converted these correlations into distances by subtracting from a value of 1 and then clustered the genomes using the “hclust” command in R using average linkage clustering. We clustered samples using the same method. Heat maps and clustering dendrograms were visualized using the heat map.2 function in the gplots package.

### Data availability.

The phylogenetic trees constructed as described here are publicly available as interactive trees at http://itol.embl.de/shared/faylward. Supplementary material (figures, tables, and data sets) and all key data products generated as part of this study are publicly available online at figshare.com/projects/Marinimicrobia_Pangenomics/30881.
